# Biocompatible Glycol Chitosan Microgels as Effective Drug Carriers

**DOI:** 10.3390/gels9050398

**Published:** 2023-05-10

**Authors:** Mehtap Sahiner, Aynur S. Yilmaz, Ramesh S. Ayyala, Nurettin Sahiner

**Affiliations:** 1 Department of Bioengineering, Faculty of Engineering, Canakkale Onsekiz Mart University Terzioglu Campus, Canakkale 17100, Turkey; sahinerm78@gmail.com; 2 Department of Chemistry, Faculty of Sciences & Arts, Nanoscience and Technology Research and Application Center (NANORAC), Canakkale Onsekiz Mart University Terzioglu Campus, Canakkale 17100, Turkey; sanemyilmazz99@gmail.com; 3 Department of Ophthalmology, Morsani College of Medicine, University of South Florida Eye Institute,12901 Bruce B Down Blvd, Tampa, FL 33612, USA; rayyala@usf.edu; 4 Materials Science and Engineering Program, Department of Chemical & Biomedical Engineering, University of South Florida, Tampa, FL 33620, USA

**Keywords:** glycol chitosan microsphere, drug delivery system, tannic acid, natural polymeric drug carriers, antioxidant activity

## Abstract

Glycol chitosan (GC) is a chitosan (CH) derivative with improved water solubility with regards to CH which affords significant solubility advantages. In this study, microgels of GC as p(GC) were synthesized by a microemulsion technique at various crosslinking ratios e.g., 5%, 10%, 50%, 75%, and 150% based on the repeating unit of GC using divinyl sulfone (DVS) as a crosslinker. The prepared p(GC) microgels were tested for blood compatibility and it was found that p(GC) microgels at 1.0 mg/mL concentration possessed a 1.15 ± 0.1% hemolysis ratio and 89 ± 5% blood clotting index value confirming their hemocompatibility. In addition, p(GC) microgels were found biocompatible with 75.5 ± 5% cell viability against L929 fibroblasts even at a 2.0 mg/mL concentration. By loading and releasing tannic acid (TA) (a polyphenolic compound with high antioxidant activity) as an active agent, p(GC) microgels’ possible drug delivery device application was examined. The TA loading amount of p(GC) microgels was determined as 323.89 mg/g, and TA releases from TA loaded microgels (TA@p(GC)) were found to be linear within 9 h and a total amount of TA released was determined as 42.56 ± 2 mg/g within 57 h. According to the Trolox equivalent antioxidant capacity (TEAC) test, 400 µL of the sample added to the ABTS+ solution inhibited 68.5 ± 1.7% of the radicals. On the other hand, the total phenol content (FC) test revealed that 2000 μg/mL of TA@p(GC) microgels resulted in 27.5 ± 9.5 mg/mL GA eq antioxidant properties.

## 1. Introduction

Chitosan (CH) is a linear polysaccharide obtained by deacetylation of the N-acetylglucosamine units of the natural polymer chitin and is suitable for a wide variety of biomedical use including drug delivery and cell imaging with its high biocompatibility, low immunogenicity, biodegradability, and low-cost properties [1]. CH also has a mucoadhesive property owing to its hydrogen bonding and hydrophobic interaction capabilities [2,3]. The cationic charges through amine groups in its backbone facilitate the chemical modification bestowing chitosan the innate assets, e.g., such as flexibility, functionality, and antibacterial properties [4,5]. However, the very low solubility of CH in aqueous solutions above pH 6 limited its’ widespread use [6]. Therefore, the protonation of primary amines in the presence of acids contributes to CH solubility [1,5]. On the other hand, glycol chitosan (GC) is a water-soluble chitosan derivative obtained by integrating a hydrophilic ethylene group rendering water solubility in a wide pH range from acidic to neutral while providing many advantages [7,8]. GC is typically derived from chitin by integrating the hydrophilic glycol group using ethylene oxide and partial deacetylation [1,9]. The molecular weight of GC varies between 20 and 250 kDa depending on the degree of deacetylation, and it is known that its molecular weight and degree of deacetylation affect the physicochemical and biological properties of GC [1,10]. For example, Park et al. reported that high molecular weight glycol chitosan nanoparticles can stay in the bloodstream longer and have better pharmacokinetic properties compared to low molecular weight ones [11]. As GC is modified, the amine groups remain unaffected and the attractive properties of chitosan such as biocompatibility, non-toxicity, biodegradability, and mucoadhesive properties are maintained [12,13,14]. Moreover, it was found that the amine groups in the backbone of the hydrophilic GC facilitate the incorporation of various hydrophobic groups and contribute to steric stabilization [15]. In addition, several studies confirmed that GC-based nanogels were safe for systemic administration [16,17]. In a recent report, GC-based nanoparticles showed a pH-dependent surface charge [18], and because of this feature, these particles can circulate at physiological pH without interacting with serum proteins [15,18]. In addition, positively charged chitosan-based particles showed a higher rate of cellular internalization and the required level of lysosomal escape after cellular uptake [19,20]. Additionally, GC has been studied for various purposes such as targeted drug delivery [21], theragnostic devices [22], and designing in vivo tumor suppression tests [23]. GC can also be modified in ways to form micro- or nano-particles as amphiphilic molecules that can self-assemble in an aqueous environment [24]. In these self-assemblies, hydrophobic moiety at the core attracts the hydrophobic molecules to reside in the inner part of the particles. This feature allows the amphiphilic particles to encapsulate the hydrophobic drugs for transportation to the intended part of the body [25]. Furthermore, a drug loading amount of up to 80% and sustained release for a week were achieved by encapsulating drugs into hydrophobically modified GC as reported in the literature [26]. GC has been modified with various hydrophobic molecules such as histidine derivatives [27], glutathione (GSH) [28], palmitic acid [29], deoxycholic acid (DOCA) [30], fullerene (C_60_) [30], and fluorescence isothiocyanate (FTIC) [30] to obtain amphiphilic GC derivatives. Duhem et al. synthesized tocol derivatives of chitosan as amphiphilic polymers and loaded them with an antifungal agent [21]. This micellar system showed sustained drug release, biocompatibility, promising bioavailability, and increased drug solubility [21]. Qu et al. prepared quaternary ammonium palmitoyl glycol chitosan (GCPQ) and loaded this sample with a steroidal and an anesthetic drug molecule separately [31]. This encapsulation resulted in higher bioavailability in the cornea [31].

Tannins are polyphenolic biomolecules that are found in a wide range of plants such as gallnuts, tea, grapes, nuts, food grains, wine, and cacao. Anionic agent tannic acid (TA) is a specific tannin that contains multiple hydroxy groups and is thus extremely soluble in water [32]. TA is a US Food and Drug Administration (FDA)-approved compound and has been used as a food additive, dispersing agent, bio-sorbent, and dietary ingredient [33]. To date, various significant features of TA have been reported in the literature such as a free radical neutralizing effect, antiviral and antibacterial properties, and homeostatic and anticancer activities [34]. TA is able to bind to other molecules by electrostatic, hydrophobic, and hydrogen bonding interactions [35]. For example, Li et al. showed that TA could promote the oxidative stability of the emulsions and convert the free radicals into more stable compounds thus inhibiting the auto-oxidation of lipids [35]. Pucci et al. stated that the encapsulation of TA could provide better cellular uptake, delay chemical deterioration, and preserve antioxidant properties in complex systems [36].

Therapeutic agents with low solubility also possess low bioavailability in the human body, thus higher doses are needed to be administered to obtain the required drug concentration in the blood [37]. On the other hand, high doses of administered drugs can cause toxicity and unwanted immune response in the body [38,39]. Therefore, it is necessary to develop carrier systems that can improve the solubility and biocompatibility of drugs and therapeutic agents, without showing any toxicity to the human body [38,40]. Recently, GC and its derivatives have been used to load various therapeutic agents such as anticancer drugs [41,42], antibiotics [43], antifungals [44], photothermal therapy agents [45,46], and certain imaging agents e.g., iron oxide [47] and gadolinium [48]. Here, p(GC) microgels were prepared via the micro emulsion method and characterized. Furthermore, blood and fibroblast cell compatibility of p(GC) microgels was determined. A polyphenol, TA was loaded into p(GC) microgels and its release in physiological pH, 7.4 (PBS) medium was monitored. It was also revealed that TA-loaded microgels (TA@p(GC)) showed antioxidant properties.

## 2. Results and Discussion

**Figure 1:**
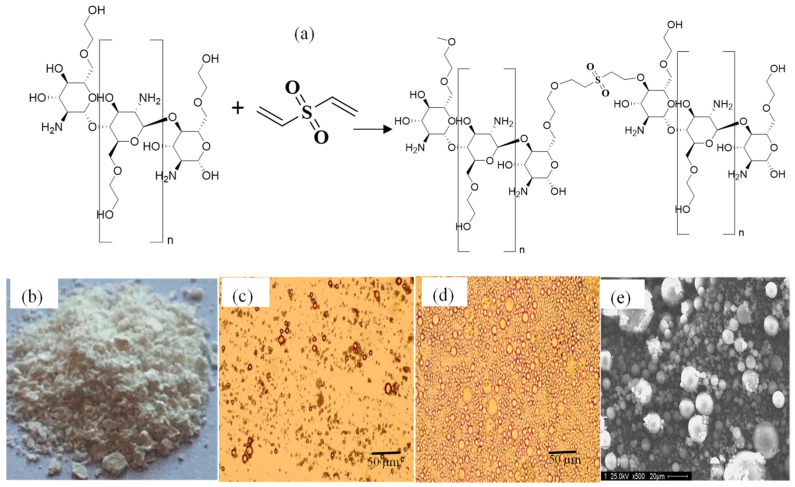
(**a**) Schematic presentation of the synthesis of p(GC) microgels, (**b**) photograph of 10% DVS crosslinked p(GC) microgels, optical microscope images of (**c**) dry, (**d**) swollen p(GC) microgels, and (**e**) scanning electron microscope (SEM) images of p(GC) microgels, respectively.

GC has reactive functional groups, including amine and hydroxy groups, which can be modified to produce a large number of derivatives [1]. In a single step, microgels of GC were synthesized via the microemulsion method by crosslinking GC units with a DVS crosslinker at different% mol ratios, e.g., 5%, 10%, 50%, 75%, and 150%. As shown in Figure 1a, p(GC) microgels are schematically represented. Figure 1b,c illustrates microscope images of dry and swollen p(GC) microgels. Figure 1b,c reveals that the microgels are highly swollen in water. As seen in Figure 1d, the p(GC) microgels are spherical in shape and the ImageJ program and SEM images were used to determine the average size of the microgels and measured as 4.5 ±2.3 µm. For the sake of less DVS incorporation into natural GC structures, 10% of DVS crosslinked p(GC) microgels were employed throughout this investigation.

**Figure 2:**
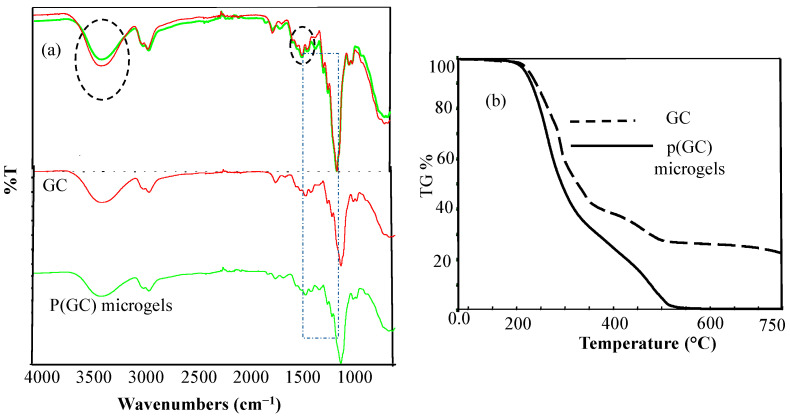
(**a**) FT-IR spectra of GC and p(GC) microgels and (**b**) thermal degradation (TG %) of GC and p(GC) microgels.

From the FT-IR spectra of GC, shown in Figure 2a, the intensity of the broadband in 3530–3130 cm^−1^ ranges is due to the stretching vibration of the -OH groups. For p(GC) microsphere, the intensity of this peak decreased due to the use of this functional group during the crosslink reaction. The peaks at 2923 and 2873 cm^−1^ wavenumbers are the O-CH_2_ symmetric and asymmetric stretching bands that belong to GC. The C-O stretching peaks of GC can be readily noticed in 1108 and 1030 cm^−1^ wavelengths. The peaks for S=O bonding are clearly detected at 1150 and 1107 cm^−1^ upon p(GC) microsphere formation by the crosslinking reaction of GC with DVS. The FT-IR spectra analysis of p(GC) microgels delivers an apparent confirmation that the crosslinking of GC chains with DVS depicts that the p(GC) microgels formation is successful.

The thermal gravimetric analysis gives the weight change quantitatively against the increasing temperature. Thermal gravimetric analysis of GC and p(GC) microgels is given in Figure 2b. The GC polymer retained 96.5% of its weight at 201 °C. At 282 °C, 50.5% of its weight remained. At 343 °C, 30% of the weight of the GC polymer stayed. On the other hand, when heating up to 498 °C, the weight of the GC polymer was increased tremendously and only 0.44% of the initial weight was retained. However, the weight loss of p(GC) microgels was lesser in comparison to GC at the same temperatures. At 201 °C, 282 °C, and 343 °C, the remaining weights of p(GC) microgels were 97.8%, 69.2%, and 43.2%, respectively. At 507 °C, there was an 83% weight loss observed (17% of the initial weight of the p(GC) microgels remained).

**Figure 3:**
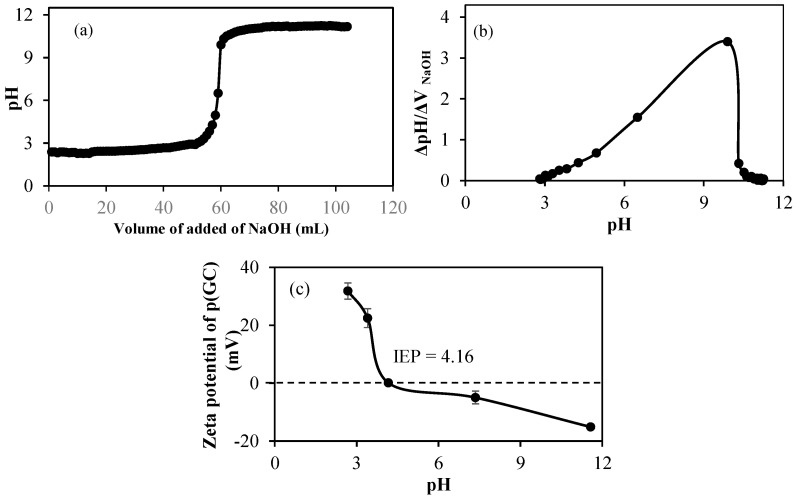
(**a**) Potentiometric titration of p(GC) microgels, (**b**) the equivalent points of the reaction calculated from the potentiometric titration, and (**c**) zeta potential measurements of p(GC) microgels at various pHs in 0.01 M KNO_3_ solutions.

The potentiometric titration of p(GC) microgels was performed in 0.01 M 50 mL KNO_3_ solution using 0.1 M NaOH and 0.1 M HCl. It was found p(GC) microgels have one pKa value at pH 9.9 from amine, as shown in Figure 3a,b as most of the hydroxy groups were used in crosslinking with DVS during microsphere formation. To determine the isoelectric point of p(GC) microgels, the zeta potentials values of p(GC) microgels in different pH solutions were measured and the results were shown in Figure 3c. It is apparent that p(GC) microgels had a positive zeta potential value of +22.5 mV at pH 3.4. Isoelectric points (IEPs) for p(GC) microgels were measured at pH 4.16, where positive and negative charges are equal.

**Figure 4:**
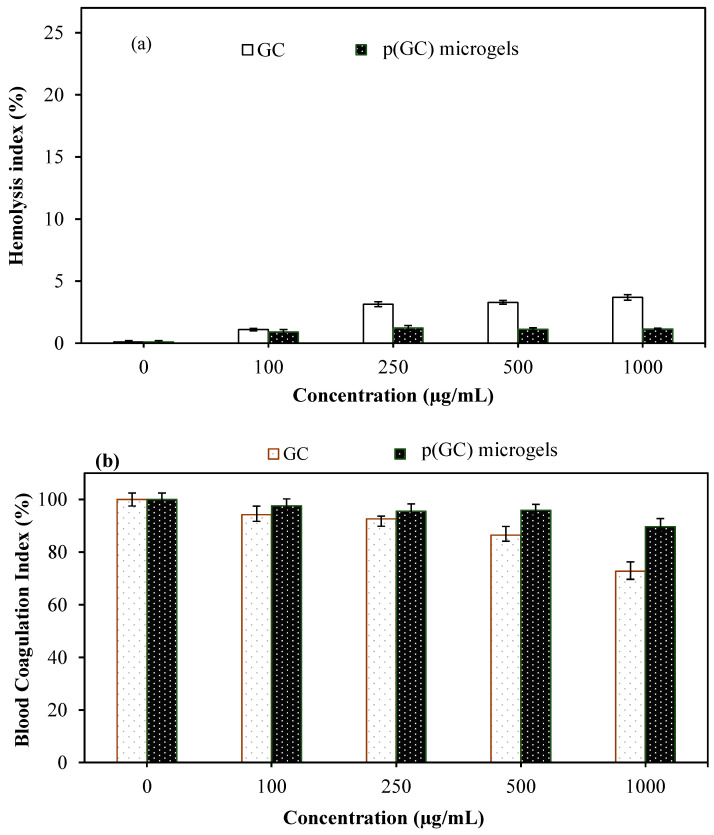
(**a**) Hemolysis index and (**b**) blood clotting index values of GC and p(GC) microgels at various concentrations.

Biomaterials, as they are frequently in contact with blood, are required to be biocompatible and should not adversely affect the normal physiologic functions of the blood cells. Therefore, the hemocompatibility of p(GC) microgels was evaluated by hemolysis and blood clotting assays, and their results are represented in Figure 4a,b, respectively. Hemolysis is a pathological condition that causes the release of the hemoglobin molecule through the disintegration of the erythrocyte cell membrane and causes many complications such as anemia, jaundice, weakness, and enlarged liver [48]. In the literature, the hemolysis ratio of materials is determined as follows: <2%: non-hemolytic, 2–5%: slightly hemolytic, and >5%: hemolytic material [49]. As shown in Figure 4a, the hemolysis ratio of GC was determined as 1.1 ± 0.1%, 3.13 ± 0.2%, 3.28 ± 0.2%, and 3.68 ± 0.2%, while the hemolysis ratios of p(GC) microgels were estimated as 0.9 ± 0.1%, 1.22 ± 0.2%, 1.12 ± 0.2%, and 1.15 ± 0.1%, at 100, 250, 500, and 1000 μg/mL concentrations, respectively. Apparently, the GC was slightly hemolytic with >3% hemolysis ratio at 250, 500, and 1000 μg/mL concentrations. On the other hand, p(GC) microgels were non-hemolytic in up to 1000 μg/mL concentrations with a <2% hemolysis ratio at 1000 μg/mL. These results clearly show that p(GC) microgels even at a 1 mg/mL concentration were compatible with human blood. The blood clotting test was another important parameter performed to investigate the interaction between materials and the clotting function of the blood.

As seen in Figure 4b, the blood clotting index values were determined as 94 ± 3%, 92 ± 1%, 86 ± 3%, and 72 ± 3% for GC, and 97 ± 3%, 95 ± 2%, 95 ± 3%, and 89 ± 5% for p(GC) microgels at 100, 250, 500, and 1000 μg/mL concentrations, respectively. It is evident that p(GC) microgels showed significantly higher blood clotting index values than GC at all concentrations studied. These results indicate that p(GC) microgels can be safely used for in vivo studies and compared to GC, p(GC) microgels are quite favorable in terms of their better hemocompatibility.

**Figure 5:**
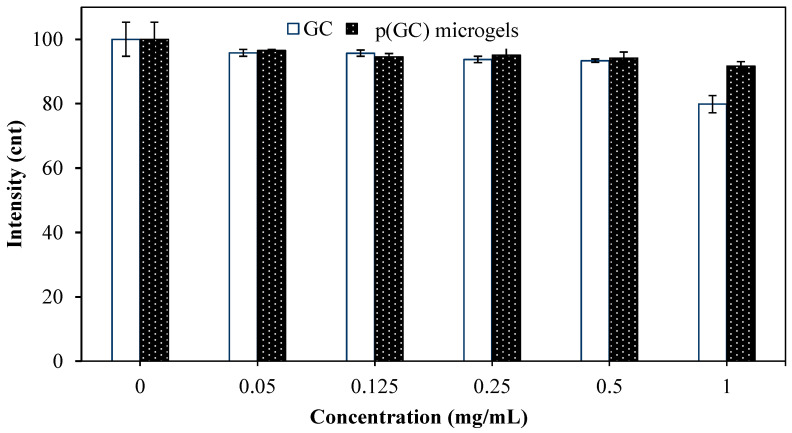
Fibrinogen interaction of GC and p(GC) microgels at different concentrations plotted as the reduction in fibrinogen emission intensity at 340 nm wavelength.

Fibrinogen is a soluble glycoprotein that plays an important role in blood clot formation and hemostasis. The change in the structure of fibrinogen and its clotting functions can be monitored by the reduction in the fluorescence properties of fibrinogen [50]. Therefore, the effect of GC-based materials, at 50, 125, 250, 500, and 1000 μg/mL concentrations on fibrinogen was investigated by Fluorescence spectroscopy and the reduction in fluorescence emissions is illustrated in Figure 5.

As seen in Figure 5, the fluorescence intensity decreased with increased concentrations of GC-based materials. Apparently, GC polymer and p(GC) microgels did not have any significant effect on the fluorescence intensity of fibrinogen up to 0.5 mg/mL concentrations. However, GC at 1 mg/mL caused a 20.12 ± 2.6% decrease in fluorescence intensity. As p(GC) microgels at the same concentration interacted with fibrinogen, the decrease in fibrinogen peak is only 8.3 ± 1%, which was quite less than that of GC polymer. Fibrinogen interaction assay results are coherent with the blood clotting index values of GC-based materials. Overall, blood compatibility studies indicate that p(GC) microgels are compatible with blood and could be safely used in blood-contacting applications.

**Figure 6:**
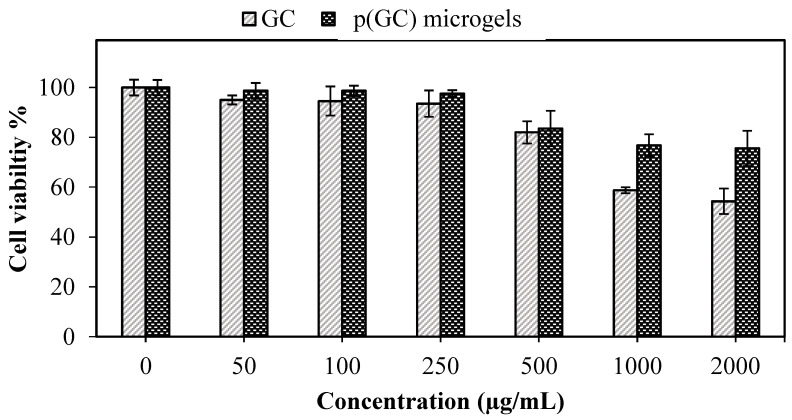
Cell viability of GC and p(GC) microgels on L929 fibroblasts at 24 h incubation time.

Mesoderm-derived fibroblasts are cells capable of transforming into any cell type in the body and are the principal cells of mature connective tissue [51]. Fibroblasts are used as model systems for the in vitro investigation of many fibrosis-based diseases and some of the inflammation, drug toxicity, and cancer-based studies [52]. Primary mouse embryonic fibroblasts are routinely used in culture studies as a support layer for the proliferation of embryonic stem cells, both for primary cell cultures and to create permanently transformed cell lines [53]. Therefore, in vitro biocompatibility of GC-based materials at various concentrations (a 2–0.05 mg/mL range) was investigated via MTT assay, and the results are demonstrated in Figure 6.

As can be seen from Figure 6, the cell viability values of both GC and p(GC) microgels decreased with the increasing concentrations. Pursuant to ISO 10993-5, percentages of cell viability above 80% are considered non-cytotoxicity, within 80–60% was weak; and below 60% was strong cytotoxicity, respectively [54]. It is apparent that GC polymer and p(GC) microgels both were biocompatible up to 500 μg/mL concentrations with >80% cell viability. GC polymer showed significant toxicity at 1000 and 2000 μg/mL concentrations with 58.6 ± 1% and 54.3 ± 5% cell viability. On the other hand, p(GC) microgels were biocompatible even at 1000 and 2000 μg/mL concentrations with 76.8 ± 4% and 75.5 ± 5% cell viability, respectively. Optic images of cell viability analysis of GC and p(GC) microgels are given in Supplementary Figure S1. As seen in Supplementary Figure S1, the fibroblasts in the control group of the MTT test (containing only DMEM growth medium) were radial, round, and spindle-shaped. GC polymer and p(GC) microgels both were biocompatible at 500 μg/mL concentrations, and their MTT test result revealed >80% cell viability. As the concentration of GC-based materials increased (the optic images seen from the top to down), the cell viability of fibroblasts decreased. GC polymer showed significant toxicity on fibroblasts at 1000 and 2000 μg/mL concentrations as seen via the disruption of cells in terms of their morphology. On the other hand, p(GC) microgels were relatively more biocompatible even at 1000 and 2000 μg/mL concentrations which were found as 76.8 ± 4% and 75.5 ± 5% cell viability, respectively, by the MTT test. The optic images of p(GC) microgels at 1000 and 2000 μg/mL concentrations are not clear due to the high concentration of the microgels. Therefore, the biocompatibility of p(GC) microgels at the highest concentrations in the MTT assay were tested for 24 h of incubation time.

As microgels of p(GC) had higher biocompatibility than GC polymer against L929 fibroblasts, it can be inferred that the toxicity disadvantage of certain active molecules can be overcome by using these microgels in their particulate forms. Biocompatibility of p(GC) microgels even at 2 mg/mL concentration with more than 75% cell viability indicates that these GC-based microgels show high potential for various biomedical applications such as the delivery of active pharmaceutical ingredients or in topical wound care.

**Figure 7:**
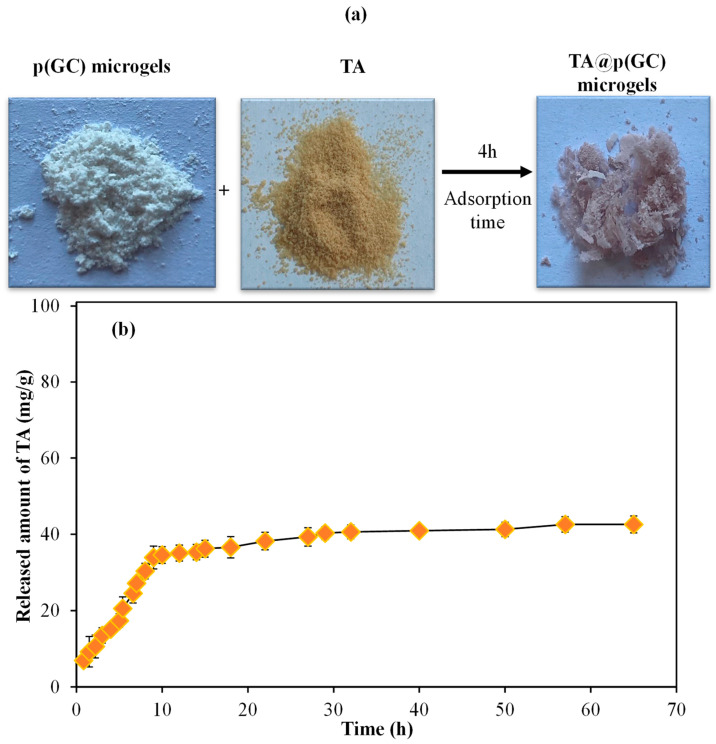
(**a**) Photographs of p(GC) microsphere, TA molecules, and TA loaded p(GC) microgels (TA@p(GC)), and (**b**) TA release profile from TA@p(GC) microgels in PBS at pH 7.4 and 37 °C.

To corroborate the drug carrier abilities of p(GC) microgels, highly antioxidant polyphenol TA was loaded into p(GC) microgels by the absorption technique. The TA loading into GC microgels and the TA release from TA@p(GC) microgels results are illustrated in Figure 7a,b, respectively.

The controllable release of TA is quite important as rapid or sustained TA release could provide valuable therapeutic effects such as antioxidant, anti-inflammatory, and antiviral activities in certain biomedical applications. TA loading amount of p(GC) microgels was measured by the absorbance values of supernatants before and after the loading process and calculated as 323.89 mg/g. As can be observed in Figure 7a, TA loading into p(GC) microgels resulted in a color change from the white color of bare p(GC) to pink/light brown color upon TA loading. As shown in Figure 7b, the release of TA from TA@p(GC) microgels is linear up to 9 h, and the release rate sharply decreased between 9 h and 57 h. The total amount of TA released was 42.56 ± 2 mg/g, which is equal to 13.14% of loaded TA content. High drug loading but relatively low drug release from p(GC) microgels suggested that these microgels can be also used for the removal of toxic substances or delivery of targeted therapy agents. The high amount of TA loading may be due to the strong interaction of cationic GC and anionic TA. The drug loading and release studies indicate that p(GC) microgels have high drug loading efficiency in addition to their linear and relatively sustained drug release profile for up to 57 h.

**Figure 8:**
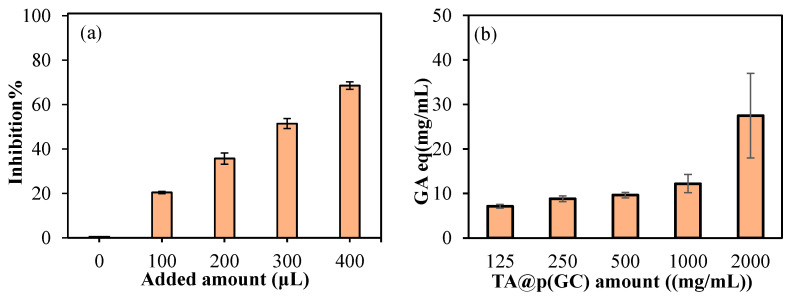
(**a**) The % inhibition values of TA release medium taken from TA@p(GC) microgels release media in the first 9 h tested and tested via TEAC assay, and (**b**) total phenol GA equivalency of TA@p(GC) microgels at different concentrations.

To determine the antioxidant capacity of TA@p(GC) microgels, two different methods were employed. In Figure 8a,b, the antioxidant results of TA@p(GC) microgels according to the TEAC and FC tests, respectively, are given. According to TEAC test, 100, 200, 300, and 400 μL of samples added into ABTS^+^ solution resulted in 20.37 ± 0.5, 35.69 ± 2.56, 51.44 ± 2.27, and 68.5 ± 1.7% inhibition, respectively. Total phenol values according to five concentrations, 125–2000 µg/mL range is given in Figure 8b. As the concentration of TA@p(GC) increases, the FC value also increased as expected. As anticipated, p(GC) particles can be used as carries even for big molecules such as TA with antioxidant properties and can also be readily used for other active agents such as antibiotics, antifungal medication as well as anticancer agents.

## 3. Conclusions

In this study, the single-step preparation of p(GC) microgels via micro emission method by crosslinking with DVS was reported. The microsphere formation was corroborated via SEM, DLS, and FT-IR spectroscopy analyses. The thermal stability of p(GC) microgels was found to be higher than that of linear GC. The prepared p(GC) microgels were spherical and a few micrometers in size (~4.5 µm) with smooth surfaces. The microgels of GC were found thermally more stable than GC. The blood compatibility of p(GC) microgels was evaluated using a hemolysis ratio and blood clotting index assay and their results were measured as 1.15 ± 0.1% and 89 ± 5%, respectively suggesting that they are hemocompatible. The cell cytotoxicity results of p(GC) microgels performed on L929 fibroblasts indicated that prepared p(GC) microgels did not induce significant toxicity for up to 2 mg/mL concentrations and showed better compatibility than linear GC. Moreover, the drug delivery efficiency of p(GC) microgels was evaluated using polyphenol, tannic acid (TA) as an active agent. P(GC) microgels loaded with TA afforded a high antioxidant activity determined by TEAC and FC antioxidant tests. The high amount of TA loading capability of p(GC) microgels (323.89 mg/g) suggests that other polyphenolics or active agents with low stability, low water-solubility, or high toxicity profile could be delivered utilizing p(GC) microgels as drug delivery vehicles for different biomedical applications.

## 4. Materials and Methods

### 4.1. Materials

Glycol chitosan (GC, powder, MW: 205.22 g/mol, MP Biomedicals, Santa Ana, CA, USA) as a polymer, divinyl sulfone (DVS, >96%, TCI, Tokyo, Japan) as a chemical crosslinker, sodium bis(2-ethylhexyl) sulfosuccinate (AOT, 96%, Sigma Aldrich, St. Louis, MO, USA) as a surfactant, and 2,2,4-trimethylpentane (isooctane, Sigma) as a solvent were used as received in p(GC) microgels preparation.

For the cell culture studies, the L929 fibroblasts (mouse connective tissue) were obtained from the SAP Institute (Ankara, Turkey). Dulbecco’s Modified Eagle’s Medium (DMEM, Pan BioNTech, Aidenbach, Germany) containing 4.5 g/L glucose, 3.7 g/L sodium pyruvate, and 0.5 g/mL L-Glutamine was purchased and used as a cell culture medium. Fetal bovine serum (FBS inactivated, Pan BioNTech, Aidenbach, Germany), and Ca/Mg-free trypsin/EDTA (Pan BioNTech, Aidenbach, Germany) were used as received. As the MTT agent, 3-(4,5-dimethylthiazol-2-yl)-2,5-diphenyltetrazolium bromide (neoFroxx, Einhausen, Germany) was purchased and used as received. Trypan blue solution (0.5%, Biological Industries, Bet-Haemek, Israel) was used for cell counting and dimethyl sulfoxide (DMSO, 99.9%, Carlo-Erba, Val-de-Reuil, France) was used for dissolving the formazan crystals.

### 4.2. Synthesis and Characterization of p(GC) Microgels

Microemulsion technique was used to prepare p(GC) microgels in 30 mL of 0.2 M AOT/isooctane media. Shortly, 0.05 g GC was dissolved in 1.5 mL of 0.2 M NaOH solution. Then, a GC solution of 0.5 mL was put into 30 mL of 0.2 M AOT/ isooctane, while stirring at 1000 rpm. Then, 5, 10, 50, 75, and 150% mol of DVS were added as a crosslinker agent, separately in the prepared GC solution. After mixing at room temperature for one hour, the obtained microgels were centrifuged at 1000 rpm for 10 min. Next, p(GC) microgels were washed with an excess amount of acetone three times, by repeated centrifugation. The microgels were dried in a vacuum oven for further analysis.

An optical microscope (Olympus, BX53, Tokyo, Japan) and scanning electron microscopy (SEM, Hitachi Ultra High-Resolution Analytical FE-SEM SU-70, Tokyo, Japan) were used to image the p(GC) microgels. As p(GC) microgels were mounted onto carbon tape-attached aluminum SEM stubs and coated with gold to a few nanometers thickness in a vacuum, SEM images were acquired at 20 kV. In order to characterize the thermal properties of p(GC) microgels, a thermogravimetric analyzer was employed (SII TG/DTA 6300, Tokyo, Japan). A ceramic pan holding about 4 mg of p(GC) microgels was heated to 50–500 °C, with the weight loss recorded at 10 °C/min and 100 mL min^−1^). The functional groups of GC and p(GC) microgels were assessed with FT-IR spectroscopy (Nicolet IS10, Thermo, Waltham, MA, USA) by taking the corresponding FT-IR spectra of the materials in the spectral range of 4000–650 cm^−1^ with a resolution of 4 cm^−1^, using the ATR technique. ImageJ was used to measure the average size of synthesized p(GC) microgels using SEM images. P(GC) microgels were titrated. For this, 50 mg of p(GC) microgels were suspended in 50 mL of 10 mM KNO_3_ solution, and 0.01 M HCl and NaOH were used to titrate the mixture. The pH range of the potentiometric titrations was 2.6 to 11.6. Zetapals zeta potential analyzer (90 plus, Brookhaven Instrument Corp., Holtsville, NY, USA) was used to measure surface charges of p(GC) spheres at different pHs.

### 4.3. In Vitro Biocompatibility of p(GC) Microgels

Biocompatibility analyses of GC-based materials were carried out on L929 fibroblasts via MTT cell viability assay as described by Mosmann [55].

Frozen L929 fibroblasts were gently thawed at 37 °C in a laminar airflow biosafety cabinet. Then, thawed cells were put in a centrifuge tube containing 2 mL of DMEM (supplemented with 10% FBS) and centrifuged at 100× *g* for 5 min. After this, the supernatant was removed and 1 mL of fresh DMEM was added, and the cell suspension was seeded into a 25 cm^2^ cell culture flask and incubated at 37 °C in a CO_2_ incubator (5% CO_2_/95% air atmosphere) for a couple of days. After the desired cell confluency was observed, the cells were detached using 2 mL of trypsin-EDTA for 5 min. The detached cell suspension was centrifuged at 100× *g*, and the cells were counted using a hemocytometer.

For the sample preparation, 20 mg of GC and p(GC) microgels were weighed and suspended in 10 mL of DMEM to obtain 2 mg/mL of initial concentrations. Then, these samples were diluted in DMEM to obtain 1000, 500, 250, 100, and 50 μg/mL concentrations of samples. L929 fibroblasts were counted and 100 μL of cell suspension containing approximately 5 × 10^3^ cells/mL were seeded onto a 96-well plate and incubated for 20–24 h at 37 °C in a CO_2_ incubator. After the incubation time, the cells were checked, old media were discarded, and immediately 0.1 mL of GC-based materials were placed onto the wells and the plate was incubated for 24 h. After this, the media were removed, and the wells were washed with phosphate-buffered saline (PBS) solution once. Then, 100 μL of 0.5 mg/mL MTT solution prepared in DMEM was added to each well and the plate was kept in the dark for 3 h. Finally, 200 μL of DMSO was added to the wells to dissolve the dark blue formazan crystals, mixed thoroughly, and kept for 20 min to homogenize the color. Optical density was measured by an ELISA plate reader (Multiskan™ FC, Microplate Photometer, Thermo Fisher Scientific, Waltham, MA, USA) at 590 nm.

### 4.4. Blood Compatibility of p(GC) Microgels

Blood compatibility analysis is one of the requirements for materials to be used in the biomedical field. Blood-contacting materials cannot cause the deterioration of red blood cells and they should be hem compatible [56]. Fibrinogen interaction, hemolysis, and blood clotting tests of p(GC) microgels were performed by following the previously reported methods [57,58]. The blood compatibility assay protocol is given in the supporting information in detail.

### 4.5. Fibrinogen Interaction of p(GC) Microgels

Fibrinogen is one of the plasma proteins synthesized in the liver. While coagulation is taking place, fibrinogen transforms into fibrin with the effect of thrombin and ionized calcium and thus mediates platelet adhesion [59]. The interactions between p(GC) microgels and fibrinogen were investigated using fluorescence spectroscopy (Thermo Scientific Lumina Spectrophotometer, Soul, South Korea). Briefly, a 0.2 mg/mL of fibrinogen solution was prepared in PBS. Then, GC-based samples were prepared in PBS at 0.1, 0.25, 0.5, 1, and 2 mg/mL concentrations. Sample solutions of 1 mL were mixed with 0.2 mg/mL of fibrinogen solution at a 1:1 ratio by volume. The excitation wavelength of 340 nm was used, and the scanning range was set between 300–450 nm. After this, 1 mL of 0.2 mg/mL fibrinogen solution mixed with 1 mL of PBS solution was used as a control and the interactions of GC-based microgels with fibrinogen were measured by the reduction in fluorescence intensity.

### 4.6. TA Delivery Studies of p(GC) Microgels

TA was loaded into p(GC) microgels through the absorption technique to assess the drug delivery potential of p(GC) microgels. For this, 20 mg of TA, an active agent which has high antioxidant activity, was dissolved in 40 mL of 1:1 volume ratio of ethanol: water solution in a falcon tube to obtain a 500-ppm concentration of TA solution. After this, 50 mg of p(GC) microgels sterilized under UV radiation (λ = 420 nm) were added to the TA solution and the tube was placed on a roller mixer (Stuart Bibby Scientific, SRT6D, London, UK) for 4 h to attain a gentle, highly efficient rolling action. After this period, the loaded microparticles as TA@p(GC) microgels were washed with a 1:1 volume of ethanol: water mixture by centrifugation at 10,000 rpm for 5 min and dried by a heat gun. The TA-loading amount of p(GC) microgels was determined from the absorbance of the TA solution before and after the loading process using a UV-Vis spectrophotometer (SP-UV300SRB, Spectrum, Quanzhou, China) at 275 nm against the corresponding calibration curves of TA prepared in the ethanol: water1:1 mixture.

The in vitro release of TA from the TA@p(GC) microgels was measured in PBS at pH 7.4 and 37 °C. For this, 25 mg of lyophilized TA@p(GC) microgels were gently put into dialysis membranes and dispersed in 1 mL of sterile PBS. Then, drug-loaded microgels containing dialysis membranes were placed into 20 mL of PBS in tubes and were incubated in a shaking bath at 37 °C during the TA release. After no more release was observed, the released amount of TA from TA@p(GC) microgels was determined via UV-Vis spectrometer using a previously created calibration curve of TA in PBS at a 275 nm wavelength. All the experiments were repeated three times.

### 4.7. Antioxidant Studies of TA Loaded p(GC) Microgels

The antioxidant activity of TA@p(GC) microgels was also investigated via the Trolox equivalent antioxidant capacity (TEAC) test [59]. For this, the drug release medium obtained from the first 9 h of the TA release was studied due to the linear TA release up to 9 h (as shown in Figure 7b). Following this, 2.5 mL of potassium persulfate with 7.5 mL of ABTS aqueous solutions were mixed to prepare an ABTS^+^ radical solution. This mixture was incubated in the dark for 14 h at 4 °C. The stock ABTS^+^ solution was diluted in PBS to obtain an absorbance value of 0.7 ± 0.02 at 734 nm wavelength. An amount of 3 mL of diluted ABTS^+^ solution interacted with 100–400 µL of the sample for 6 min. At the end of the 6 min, the values showing a 20−80% reduction of the blank absorbance at 734 nm were chosen. The results are given as %inhibition values with standard deviations.

According to the literature, the total phenol content (FC) of TA@p(GC) microgels was tested [60]. In brief, 2000 µg/mL of TA@p(GC) microgels were prepared and mixed overnight at 500 rpm. Several dilutions of this suspension solution were performed, ranging from 1000 to 500 to 250 to 125 µg/mL. In a 96-well plate, 20 µL of sample solution was placed, and 125 µL of FC solution was added. Afterward, 100 µL of 0.7 M Na_2_CO_3_ aqueous solution was added to the medium, and the medium was incubated in the dark for two hours. After that, the solutions were read at 760 nm with a microplate reader (Thermo Scientific, Multiskan SKY, Waltham, MA, USA). Using gallic acid (GA) as a calibration curve, the values were given as GA equivalents.

## Data Availability

The data presented in this study are available on request from the corresponding author.

## Supplementary Materials

The following supporting information can be downloaded at: https://www.mdpi.com/article/10.3390/gels9050398/s1, Figure S1: Optic images of cell viability of L929 fibroblasts in the presence of Glycol chitosan and Glycol chitosan microgels.
