# Supplementary material for: Biocompatible Glycol Chitosan Microgels as Effective Drug Carriers
Source: Gels. 2023 May 10;9(5):398. doi: 10.3390/gels9050398 (PMC10217477; doi:10.3390/gels9050398)
Supplement: Supplementary file 1 [file gels-09-00398-s001.zip › gels-2355059-supplementary.pdf]

Supporting Information

Biocompatible Glycol Chitosan Microgels as Effective Drug Carriers

Mehtap Sahiner [1], Aynur S. Yilmaz [2], Ramesh S. Ayyala [3] and Nurettin Sahiner [2,3,4,*]

[1] Department of Bioengineering, Faculty of Engineering, Canakkale Onsekiz Mart University Terzioglu Cam-pus, Canakkale 17100, Turkey; sahinerm78@gmail.com
[2] Department of Chemistry, Faculty of Sciences & Arts, Nanoscience and Technology Research and Appli-cation Center (NANORAC), Canakkale Onsekiz Mart University Terzioglu Campus, Canakkale 17100, Turkey; sanemyilmazz99@gmail.com
[3] Department of Ophthalmology, Morsani College of Medicine, University of South Florida Eye Institute, 12901 Bruce B Down Blvd, Tampa, FL 33612, USA; rayyala@usf.edu
[4] Materials Science and Engineering Program, Department of Chemical & Biomedical Engineering, University of South Florida, Tampa, FL 33620, USA
* Correspondence: sahiner71@gmail.com or nsahiner@usf.edu

1. Blood compatibility assay of GC based materials

Fresh human blood was drained from healthy volunteers and transferred to tubes containing anticoagulant. All solutions were adjusted to 37 °C before the test. For the hemolysis assay, 10, 5, 2.5, 1 mg of GC was dissolved and 10, 5, 2.5, 1 mg of p(GC) microspheres were suspended in 9.8 mL of 0.9% NaCl solution to obtain various concentration of samples. Fresh blood was diluted with 0.9% aqueous NaCl to prepare 1:1.25 (v:v) ratio of blood: 0.9% aqueous NaCl solution and 0.2 mL of the diluted blood was transferred to the tubes containing GC or p(GC) microspheres and kept in a water bath at 37.5 °C for 1 h. Separately, 0.2 mL of the diluted blood was added into 10 mL of 0.9% aqueous NaCl solution and DI water as a negative and positive control, respectively, and kept in the same conditions. After the incubation period, the tubes were centrifuged at 100*g* for five minutes and the absorbance values for the supernatants were measured at 542 nm with UV-Vis spectroscopy (SP-UV300SRB, Spectrum, China). The hemolysis ratio of the GC based materials was evaluated using Equation 1.

$$Hemolysis\ ratio\ \% = \frac{(A_{material} - A_{negative})}{(A_{positive} - A_{negative})}\ x\ 100 \qquad (1)$$

Here "$A_{material}$" is the absorbance value of the blood solution interacted with materials in 0.9% aqueous NaCl solution. "$A_{negative}$" and "$A_{positive}$" are the absorbance values of the blood solution without materials in 0.9% aqueous NaCl solution and in DI water, respectively. All assays were carried out three times and the results are given with standard deviations.

The impact of p(GC) microspheres on the coagulation activity of the blood was also assessed via the blood clotting assay. Briefly, 64 µL of 0.2 M $CaCl_2$ aqueous solution was mixed with 810 µL of blood containing EDTA and immediately 270 µL of this blood was covered with 10, 5, 2.5, 1 mg of GC and p(GC) microspheres. After 10 min, 10 mL of DI water was gently added into the tubes and centrifuged at 100*g* for 1 minute. Then 10 mL of supernatant solution containing non-clotting blood was taken from the tube and diluted with 40 mL of DI water. Separately, 250 µL of the blood containing EDTA was dispersed in 50 mL of DI water as a control. The tubes were incubated at 37.5 °C in a water bath for 1 h and the absorbance values of the supernatants were

measured at 542 nm by using UV-Vis spectroscopy. The blood clotting index of p(GC) microspheres was evaluated from Equation 2.

$$Blood\ Clotting\ Index = (A_{material}/A_{control})\ x\ 100 \quad (2)$$

Here "$A_{material}$" is the absorbance value of the blood solution interacted with the p(GC) microspheres and "$A_{control}$" is the absorbance value of the blood solution without the prepared material as a control. All assays were performed three times and the results are given with standard deviations.

2. Cytotoxicity test of glycol chitosan and glycol chitosan particles

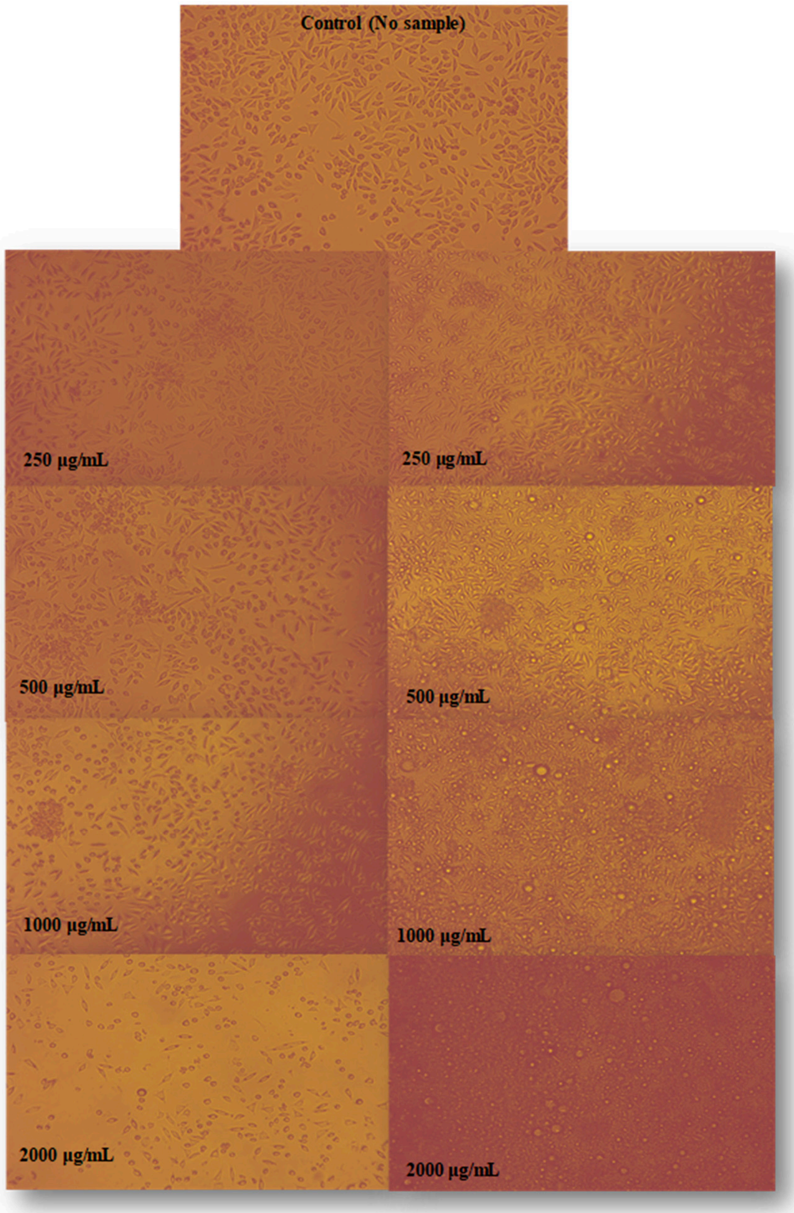

Figure S1. Optic images of cell viability of L929 fibroblasts in the presence of Glycol chitosan and Glycol chitosan microgels

As seen in Supp. Fig. S1, fibroblasts in the control group of the MTT test (containing only DMEM cell growth medium) were stellate, plump, and spindle shaped. According to this, GC polymer and

p(GC) microgels both were biocompatible at 500 μg/mL concentrations and their MTT test result revealed >80% cell viability. As the concentration of GC-based materials increased, (the optic images seen from the top to down), cell viability of fibroblasts decreased. GC polymer showed significant toxicity on fibroblasts at 1000 and 2000 μg/mL concentrations which can be clarified by the disruption of cells in terms of their morphology. On the other hand, p(GC) microgels were biocompatible even at 1000 and 2000 μg/mL concentrations which were found as 76.8±4% and 75.5±5% cell viability, respectively, by the MTT test. The optic images of p(GC) microgels at 1000 and 2000 μg/mL concentrations are not clear due to high concentration of these microgels. Therefore, biocompatibility results of p(GC) microgels at the highest concentrations were expressed based on the MTT assay results obtained at 24h incubation.
